# Combinations of Drugs in the Treatment of Obesity

**DOI:** 10.3390/ph3082398

**Published:** 2010-07-27

**Authors:** Bruno Halpern, Eduardo S. L. Oliveira, André M. Faria, Alfredo Halpern, Maria Edna de Melo, Cintia Cercato, Marcio C. Mancini

**Affiliations:** 1 Obesity & Metabolic Syndrome Group, Discipline of Endocrinology & Metabolism, Hospital das Clínicas, Faculty of Medicine, University of São Paulo, Av. Dr. Enéas de Carvalho Aguiar, 255, 7º andar, sala 7037, 05403-000 - São Paulo, SP, Brazil; E-Mails: brunohalpern@hotmail.com (B.H.); eduardolorena@uol.com.br (E.S.L.O.); andremfaria@hotmail.com (A.M.F.); alfredo.halpern@netpoint.com.br (A.H.); ccercato@netpoint.com.br (C.C.); 2 Laboratory of Carbohydrates and Radioimuneassay/LIM-18, Faculty of Medicine, University of São Paulo, Av. Dr Arnaldo, 455 01246-903 - São Paulo, SP, Brazil; E-Mail: medna@usp.br (M.E.de M.)

**Keywords:** drug combination, phentermine, fenfluramine, ephedrine, caffeine, sibutramine, orlistat, metformin, bupropion, naltrexone, zonisamide, topiramate, pramlintide, metreleptin, leptin, glucagon-like peptide-1, peptide YY, 5-hydroxytryptophan, carbidopa, noradrenergic drugs, antidepressants

## Abstract

Obesity is a chronic disease associated with excess morbidity and mortality. Clinical treatment, however, currently offers disappointing results, with very high rates of weight loss failure or weight regain cycles, and only two drugs (orlistat and sibutramine) approved for long-term use. Drugs combinations can be an option for its treatment but, although widely used in clinical practice, very few data are available in literature for its validation. Our review focuses on the rationale for their use, with advantages and disadvantages; on combinations often used, with or without studies; and on new perspectives of combinations being studied mainly by the pharmaceutical industry.

## 1. Introduction

The prevalence of obesity, defined as a body mass index (BMI) ≥ 30 kg/m^2^, has increased dramatically over the last four decades, reaching almost one third of the populations of developed countries of North America and Europe, and with overweight levels (BMI ≥ 25 kg/m^2^) of more than 40% of the population in Brazil [1]. This is associated with a significant increase of comorbidities such as type 2 diabetes mellitus (T2DM), hypertension, cardiovascular disease, obstructive sleep apnea, musculoskeletal diseases and neoplasias [2]. Studies have shown that even small weight losses of 5–10% of body weight, independently of how they were achieved, are associated with an improvement of the cardiovascular risk profile and a lower incidence of T2DM [3]. 

In recent years, obesity has been considered a metabolic and neuroendocrine disease, which results from the interface between an obesogenic environment and an intrinsic genetic predisposition. Despite this, lifestyle changes proposed in the treatment of obesity are essential and may be effective, but the rates of long term success tend to be low.

In this context, obesity pharmacotherapy plays an important role. The major limitations of obesity pharmacotherapy are the modest efficacy seen with the medications currently available and the limited availability of medications (orlistat and sibutramine) for long term treatment [4,5], although there might still be a place for catecholaminergic drugs as an option of second-line treatment [6].

In a recent meta-analysis, it was shown that both sibutramine and orlistat alone lead to placebo-subtracted weight loss on average of 3–5%, although the proportion of individuals with weight losses greater than 5% was of 54 and 55%, compared with 27 and 33% for placebo [4]. When weight losses of over 10% were analyzed, 26 and 28% were found with the medications against 10 and 14% with the placebo [4]. However, less than 10% of the patients continued using the medications after 1one year [7]. Slowing of the weight loss rate with failure to reach the expected weight is one of the factors which lead to loss of motivation and abandonment of treatment, besides the discomfort of chronic use and possible side effects.

This modest efficacy contrasts with the maintained weight loss and the low rates of therapeutic failure achieved in patients submitted to bariatric surgery, resulting in a significant reduction of comorbidities and increased life expectancy [8]. 

In addition, sibutramine was taken off the European market in 2009 due to the results of the *Sibutramine Cardiovascular OUtcomes Trial* (SCOUT) [9] which showed increased cardiovascular mortality in patients using the medication in the group with prior cardiovascular disease. Although still available in other countries, the drug should not be recommended for this subgroup of patients.

Therefore, at the same time new drugs are being tested, it seems attractive to use different strategies, such as combination of medications. Considering that obesity is a multifactorial disease, with environmental factors linked to genetic factors, it is expected that drug monotherapy may result in unsatisfactory outcomes. In analogy to the treatment of hypertension, where a combination of antihypertensive agents with different mechanisms of action is widely used to control blood pressure, obesity treatment using combinations of medications, working synergistically or in parallel [10], can result in a higher chance of weight loss and a reduction in the dropout rates.

Although little data exists for the use of combinations, in a questionnaire answered by obesity specialists in the US, 85% prescribed combinations of drugs, and 65% of them prescribed combinations not approved for obesity treatment. This result is very relevant and shows us how important is to study and publish more about this issue [11]. 

In this review, anti-obesity drug combinations in general will be discussed, covering those already studied, those still awaiting final results to be released and also those which are used empirically in clinical practice. It is worth noting that with regard to the drugs in initial studies, much of the information and clinical outcomes are from the pharmaceutical companies own websites, since they have not been yet submitted to peer-review in medical journals. Therefore, these results are subject to potential conflicts of interest. Table 1 and Table 2 list all the combinations covered in this review.

**Table 1:** Established mechanisms of action of combinations of drugs for the treatment of obesity.

Drug Combination	Mechanisms of Action
**Bupropion¹ + Naltrexone²**	¹Inhibits norepinephrine and dopamine reuptake (stimulates POMC) and ²blocks opioid receptor (prevents auto-inhibition of β-endorphine on POMC pathway)
**Bupropion¹ + Zonisamide²**	¹Inhibits norepinephrine and dopamine reuptake (stimulates POMC) and ²rises serotonin and dopamine levels (stimulates POMC and inhibits AgRP)
**Phentermine¹ + Topiramate²**	¹Increases norepinephrine release and ²unknown mechanism—in animal models reduces appetite and increases thermogenesis and fat oxidation
**Phentermine¹ + 5-HTP/Carbidopa²**	¹Increases norepinephrine release and ²inhibits peripheral conversion of 5-HTP to serotonin, reducing chance of nausea, more 5-HTP reaches the CNS and is transformed to serotonin
**Pramlintide¹ + Metreleptin²**	¹Amylin analog—increases satiety and reduces food intake, and ²reverses effects of weight loss in energy expenditure; synergistic action with pramlintide in anorexigenic signaling in CNS
**Pramlintide¹ + Sibutramine²**	¹Amylin analog—increases satiety and reduces food intake, and ²inhibits serotonin and norepinephrine reuptake; synergistic action with pramlintide in anorexigenic signaling in CNS
**Pramlintide¹ + Phentermine²**	¹Amylin analog—increases satiety and reduces food intake, and ²increases norepinephrine release; ²synergistic action with pramlintide in anorexigenic signaling in CNS

POMC: Proopiomelanocortin; AgRP: Agouti-related peptide; 5-HTP: 5-Hydroxytryptophan; CNS: Central nervous system.

**Table 2:** Briefing of available data of off-label combinations of drugs for the treatment of obesity.

Off-Label Combination of Drugs	Briefing of Available Data
**Orlistat + Sibutramine**	Few studies—weight loss similar to sibutramine alone One clinical series with good results
**Orlistat +Metformin**	One study—no benefit in association in non insulin resistant women
**Phentermine + 5-HTP/Carbidopa**	Widely used—no controlled studies
	Clinical series suggests good results
**Orlistat and noradrenergic drugs**	No studies
**Orlistat and topiramate**	No studies. Safe to use in patients with other indications of topiramate (migraine, epilepsy, eating disorders)
**Orlistat and antidepressants**	No studies. Safe to use in patients with indications of antidepressants use (anxiety or eating disorders, depression)
**Sibutramine and topiramate**	No studies. Both acts in CNS, via different mechanisms—no data about safety

CNS: Central nervous system; 5-HTP: 5-Hydroxytryptophan.

## 2. Pros and Cons of Combined Medication Therapy

The possible advantages of combination drug therapy are as follow [12]:

(1) Increased efficacy of treatment through synergic or additive action;
(2) Promotion of endogenous synergism;
(3) Medications with different pharmacodynamic actions and that act on different pathways may “beat” the compensatory mechanisms involved in homeostasis and thus prevent or delay the formation of the *plateau* during the effort to lose weight or combination of medications with opposite side effects, such as a medication associated with insomnia with another to drowsiness, could increase the tolerability of the treatment.
(4) Combination of low doses could reduce the risk of adverse effects;

The disadvantages include:

(1) Increased incidence of side effects, due to additive action and interaction of medications;
(2) Inflexibility of dosing in combo drugs prescribed in a single agent;
(3) Cost of treatment.

## 3. History of Combined Medical Therapy

Some drug combinations have already been studied in the past and stopped being used. It is worth remembering that the suspension of the commercialization of these combinations was on account of side effects of one of their components and not due to any negative interaction between the drugs. 

### 3.1. Phentermine and Fenfluramine

Phentermine is a catecholaminergic substance that increases the release of noradrenaline in the central nervous system (CNS), available for obesity treatment in the United States of America (USA) since 1959 (in doses of up to 37.5 mg/day) [13,14]. Weight loss is the result of the reduction of caloric ingestion through reduced appetite, with previous randomized studies showing reductions of 5 to 15% of initial weight in up to 60% of the patients after 16 to 36 weeks of treatment [13,14].

Fenfluramine raises the synaptic levels of serotonin in the CNS by stimulating the secretion and inhibiting the reuptake of serotonin. Dexfenfluramine is a highly selective D-isomer, which also leads to increased levels of serotonin and stimulates the hypothalamus [15]. A randomized study performed with dexfenfluramine showed significantly greater weight loss than placebo [16].

The combination of fenfluramine and phentermine resulted in a reduction of side effects and increased efficacy, even in lower doses, when compared to use of either medications by itself [17]. This combination was widely used before fenfluramine was banned from the market in 1997 due to increased risk of valvulopathy and primary pulmonary hypertension [18].

### 3.2. Ephedrine, Caffeine and Acetylsalicylic Acid

Ephedrine is a nonspecific beta-adrenergic agonist which promotes weight loss through increasing thermogenesis. Cafeine and acetylsalicylic acid reduce the degradation of ephedrine, potentiating its action [19]. The use of ephedrine combined with caffeine and/or acetylsalicylic acid is approved as a combination therapy for the treatment of weight loss in many countries [20]. In 1977, in Denmark, more than 70,000 patients used the combination of ephedrine with caffeine and even phenobarbital, which was withdrawn from the “formula” after reports of serious skin reactions. The combination of ephedrine and caffeine (20/200 mg) three times a day (tid) for 24 weeks proved to be more effective for weight loss than placebo or monotherapy with its components [21]. Many studies however have raised concerns about the safety of ephedrine and its derivatives, and the commercialization of ephedra alkaloids was banned in the United States in 2004 due to adverse cardiovascular and cerebrovascular effects [23,24], although ephedrine is still commercialized for use under rigorous monitoring. Around 1.4% of specialist physicians in US still use ephedrine with caffeine for treatment of obesity [11].

## 4. Combinations Currently in Use and Being Studied

### 4.1. Sibutramine and Orlistat

Sibutramine and its metabolites act in the CNS inhibiting the reuptake of serotonin and norepinephrine, resulting in reduction of appetite, increase in satiety and possible increase of thermogenesis [9,15,24].

Orlistat is a synthetic hydrogenated derivative of lipstatin which partially inhibits the gastric lipase, pancreatic lipase and carboxyl ester lipase enzymes. These enzymes work hydrolyzing the dietary triglycerides into fatty acids and monoglycerides, which are subsequently absorbed by the mucosal cells of the gastrointestinal (GI) tract. Orlistat reduces the absorption of ingested fat by 30%, increasing its excretion in the feces [25]. 

Since the mechanisms of action of these two medications are very distinct, their combined use was evaluated in some studies. In obese women who had already been using subutramine for one year, the combination of orlistat *versus* placebo for 16 weeks did not result in greater weight losses [26]. 

Two studies were conducted at different centers in Turkey, with similar results [27,28]. In a randomized open-label study of 12 weeks, treatment with diet alone (n = 19) was compared with diet combined with the use of sibutramine (n = 22), orlistat (n = 25) and sibutramine combined with orlistat (n = 20) [27]. The combination of sibutramine and orlistat proved more effective in the reduction of BMI in relation to monotherapy with orlistat (p < 0.001), but not significantly superior to sibutramine alone. Placebo-subtracted weight loss of the combined therapy was 6.5% (13.4% combination *vs.* 6.9% placebo). It is noteworthy that this clinical trial, like many others in the obesity field, had a run-in phase which required weight loss of 2.5 kg, a method highly criticized by authors in the field [29].

Another study involving 89 obese women, showed a mean weight loss of 5.5%, 10.2% and 10.6% with orlistat alone, sibutramine alone and combined therapy, respectively, once again with no statistical difference between the latter two groups. Despite these disappointing results, around 14% of obesity specialists reported to use this combination in clinical practice [28].

A large clinical series done by our group was developed to evaluate the efficacy of the combination of sibutramine and orlistat in 446 individuals for six months, with concomitant dietary counseling [10]. Weight loss in three and six months was 9.9 and 13.4% for women and 8.7 and 12.3% for men. After six months, 88.7% of the patients that finished the study had lost more than 5% of baseline weight and 66% had lost more than 10%. The rate of discontinuation was 37%. Although an open-label study, we consider not only the number of patients enrolled to be expressive but also the proportion of patients with significant weight loss. 

In summary, studies of combinations that are safe and widely used in clinical practice worldwide are still very scarce. It is our belief that this combination appears to provide satisfactory results, although this was not confirmed in randomized controlled studies. In contrast with our opinion, some specialists in the obesity field believe that concomitant use of drugs acting on the gastrointestinal tract with centrally acting drugs does not result in additional weight compared to the latter by itself [30].

### 4.2. Orlistat/Metformin

Only one study evaluated orlistat in combination with metformin [31], an insulin-sensitizing drug known to modestly reduce weight in insulin resistant patients [32]. Fifty seven obese women without glucose intolerance were randomly assigned to receive orlistat, 360 mg/d, or orlistat, 360 mg/d, plus metformin, 1,700 mg/d. No differences were observed with respect to weight loss or insulin resistance indexes. At present, metformin for weight loss should only be used in patients with carbohydrate metabolism disturbances. 

### 4.3. Bupropion/Naltrexone

The combination of bupropion with naltrexone (Contrave^®^) has been studied for some years by the pharmaceutical company Orexigen Therapeutics Inc. as a potential agent in the treatment of obesity [33]. Bupropion is a reuptake inhibitor of dopamine and norepinephrine currently approved for the treatment of depression and as a supporting aid for smoking cessation, with effective yet modest results in weight loss [34]. 

Naltrexone is an opioid receptor antagonist, initially approved as a treatment for opiate addiction, and later, for alcohol dependence. Studies carried out on a small number of patients using naltrexone in monotherapy as an anti-obesity agent proved frustrating, with little or no reduction in body weight [35].

Despite these drugs having a modest efficacy when used in isolation, current understanding of the complex processes of central and peripheral appetite regulation [36,37] enabled the hypothesis of a possible synergistic effect of their combination. The increase of dopamine and norepinephrine levels mediated by bupropion stimulates the neural activity of proopiomelanocortin (POMC). POMC neurons, which located in the arcuate nucleus of the hypothalamus, when stimulated leads to the formation of other peptides. POMC is cleaved into α-MSH and β-endorphin. Beta-endorphin exerts negative feedback inhibition on POMC activity and increases food intake. Naltrexone, by inihibiting opioid receptors, releases POMC neurons from the feedback inhibition exerted by beta-endorphin. The increase of α-MSH leads to the activation of MC4R and of the secondary signaling pathways which results in increased energy expenditure and reduction of appetite [38,39].

Additionally, some studies on rats have demonstrated that the ingestion of palatable foods (e.g., candies) leads to increased β-endorphin levels in the hypothalamus. With this data, it is assumed that the release of opioids has a role in the expression of food palatability, and *vice-versa* [35]. Some anecdotal reports in humans in the 1980s suggest that naltrexone, although not directly affecting hunger, decreases the feelings of pleasure associated with the act of eating, which culminates with reduced ingestion of food [35]. The synergistic effect of this combination was proven in studies in rodents, in which a reduction in food intake was shown both in lean and diet-induced obesity mice, compared with both drugs in monotherapy and placebo [39].

A double-blind phase 2 randomized study with a 24-week duration, with 419 patients, compared three different doses of immediate release (IR) naltrexone (16, 32 and 48 mg/d), in combination with bupropion sustained release (SR), 400 mg/d, with placebo, bupropion SR, 400 mg/d, and naltrexone IR, 48 mg/d, in monotherapy [40]. 

Patients receiving bupropion alone or the three doses of the combination were followed for an additional 24 week-extension. In 24 weeks the weight loss by intent-to-treat analysis (ITT) was 5.4%, 5.4% and 4.3% for the combination of bupropion with naltrexone, 16 mg (NB16), 32 mg (NB32) and 48 mg (NB48) respectively, against 2.7% for bupropion, 1.2% for naltrexone and 0.8% for placebo. The lower weight loss seen in the patients receiving NB48 was justified by the higher dropout rate (63%) due to side effects in this group. After 24 weeks, there was no sign of a plateau in any of the three combination groups, and in the final analysis of 48 weeks, the NB32 combination reached a higher success rate with an average weight loss of 6.6%. It was also found in other phase 2 studies that naltrexone SR resulted in fewer side effects compared to the IR formulation [39].

Based on these results, phase 3 studies were designed accordingly, using naltrexone SR in doses of 16 and 32 mg/d and bupropion SR, 360 mg/d, on a twice-daily basis (bid). The COR-I trial randomized 1.742 patients for NB16, NB32 or placebo, with four weeks of titration of medication and a duration of 56 weeks [33]. The placebo-subtracted weight loss (PSWL) by ITT analysis was 3.7 and 4.8% for NB16 and NB32, respectively. The COR-II trial tested NB32 in 1,001 patients, against 495 that received placebo [33]. The results were similar to those of COR-I, with PSWL by ITT analysis of 5.2%. The proportion of patients with ≥ 5% reduction of initial weight compared to placebo was also similar in COR-I (48 *vs*. 16%) and in COR II (56 *vs*. 17%).

Dropout rates were high for both studies (46 and 51%, no difference compared to placebo), with around half of the cases being directly related to side effects. The most commonly observed side effect was nausea, around 30%, compared to 5 and 6% in the placebo group. The incidence occurred mainly in the initial weeks of treatment, still within the titration phase, with a tendency to fall after the fourth week. Other side effects significantly more common than placebo, in order of frequency were: Constipation, headache, dizziness, vomiting and dry mouth. There was no significantly higher incidence of adverse effects on the cardiovascular system, nor in relation to depressive disorders or suicidal ideation. BP of patients using the combination remained unchanged after 56 weeks, with a slight decrease of 2 mmHg in the placebo group, and a negligible increase of one beat per minute in the heart rate of the treated patients. Three serious events were reported, possibly related to the use of one component of the combination: seizure (one), paresthesias (one) and palpitations with dyspnea and anxiety (one).

### 4.4. Bupropion/Zonisamide

Although in earlier stage of development, the combination of bupropion/zonisamide also seems promising as an anti-obesity drug. Zonisamide has been used as an antiepileptic drug in Japan since 1989, being approved for such use in the United States (USA) and in some European countries in the early 2000s. In some short-term studies of treatment of epileptic patients with zonisamide, weight loss was observed as a side effect [41]. The mechanism of action for the antiepileptic activity of this medication is not totally clear, but it is believed that it is linked of sodium and calcium channels blockage. *In vitro* evidence has also shown that this drug leads to an increase in serotonergic and dopaminergic activity. It is assumed that this mechanism explains, at least in part, its effects as a weight-reducing agent. Another property of zonisamide is the relatively weak inhibition of carbonic anhydrase activity. This evidence highlights previous reports of taste alteration with other carbonic anhydrase inhibitors (e.g., topiramate, acetazolamide), thus possibly leading to an anorectic effect. 

A double-blind randomized pilot study with 60 patients, showed after 16 weeks a weight loss of 6.0% *vs*. 1.0% of for zonisamide in monotherapy (usual dose of 400 mg/d) and placebo, respectively, in association with a hypocaloric diet with a 500 kcal/d deficit [42]. An open-label extension of the study for additional 16 weeks with 37 patients showed continuation of these findings with a final result of 9.4% *vs*. 1.8% reduction of the initial weight. The drug was well tolerated in this study, with fatigue being the only side effect reported with a higher incidence in the active group than in the placebo. As seen in other studies of epileptic patients, there was a significant increase of serum creatinine of 0.13 mg/dL in the treated group compared to 0.03 mg/dL in the placebo group. In a subsequent study with obese patients with binge eating disorder (BED), zonisamide (in doses up to 600 mg/day) also proved effective both in reducing weight and in controlling bingeing episodes [43].

However, this study raised concerns regarding its tolerability, since eight out of 30 patients using the drug abandoned the study due to side effects, which included psychological complaints (two), cognitive changes (two) and even accidental injury with bone fracture (two). 

Orexigen Therapeutics Inc. also decided to register the patent of zonisamide with bupropion (Empatic^®^). In the phase 2 studies conducted so far, it has chosen to test lower doses of zonisamide (120 and 360 mg/d) with doses of bupropion the same as of Contrave^®^ (360 mg/d), bid [33]. A 24-week long study of 320 patients showed by ITT analysis loss of 7.5%; 6.1% and 1.4% of initial weight for the combination of bupropion with zonisamide, 360 mg/d (ZB360), zonisamide, 120 mg/d (ZB120), and placebo, respectively (PSWL of 6.1% for ZB360 and 4.7% for ZB120) [33]. The most common side effects shown were headache (13–15%), insomnia (12–15%) and nausea (8–12%). No serious adverse effects were reported on cognitive function, depression, suicidal ideation or anxiety. Currently, this combination is awaiting completion of phase 3 studies. 

### 4.5. Phentermine /Topiramate

Topiramate is a medication originally approved for the treatment of epilepsy, which currently is also widely prescribed for migraine prophylaxis. Some studies in the early 2000s proved the efficacy of this drug to reduce the weight of obese patients (in tested doses of 64 to 384 mg/d) [44], a fact previously observed in clinical trials involving normal and obese epileptic patients. The efficacy of the drug increased very mildly with doses greater than 192 mg/d, and with the inconvenience of increased side effects. One point of interest observed in these studies was the continuation of weight loss for over a year of treatment, without the plateau usually seen after around six months with the use of other existing drugs. [44]. Topiramate was also tested in obese patients with BED, with good results for weight loss and reduction of bingeing scores [45].

However, despite showing promising efficacy, the enthusiasm with the drug diminished considerably due to the elevated incidence of side effects, such as paresthesias, memory changes, concentration impairment and mood swings. While it is known that it exerts its anti-epileptic activity through blocking sodium and calcium channels, enhancing the action of the GABA and carbonic anhydrase inhibition, its mechanism of action as a weight-loss agent is still poorly understood. In animal models, topiramate reduces both appetite and interferes with the efficiency of energy use, by increasing thermogenesis and fat oxidation, probably through stimulation of lipoprotein lipase in brown adipose tissue and skeletal muscle [44].

By combining lower doses of topiramate with phentermine, which act by different pathways, the pharmaceutical company Vivus Inc. aimed to increase the positive results and minimize the chance of side effects. Supposedly, the light stimulative effect of phentermine on the CNS could overcome the negative effects of topiramate on memory and cognition. Three doses of this combination (Qnexa^®^) were tested: low (phentermine IR 3.75 mg/topiramate SR 23 mg), medium (phentermine IR 7.5 mg/topiramate SR 46 mg) and high (phentermine IR 15 mg/topiramate SR 92 mg). The EQUATE phase 2 study evaluated 776 patients for a period of 28 weeks (including a 4-week titration phase), where the patients were randomly selected for placebo, phentermine or topiramate in monotherapy in the medium and high doses and Qnexa^®^ in medium and high doses [46]. At the end of the study, patients receiving Qnexa^®^ both in medium and high doses lost significantly more weight than phentermine or topiramate high dose in monotherapy or placebo, proving, therefore, the synergistic effect of the combination (ITT analysis, high dose Qnexa^®^: −9.2%; medium dose Qnexa^®^: −8.5%; topiramate 92 mg: −6.4%; phentermine 15 mg: −6.1%; placebo: −1.7%).

The results from phase 3 studies, with 56 weeks of duration, were recently reported. The EQUIP trial, involving 1.267 obese patients with BMI > 35 kg/m^2^, showed by ITT analysis reduction of 11.0%, 5.1% and 1.6% for Qnexa^®^ high dose, low dose and placebo, respectively (PSWL of 9.4% for high dose and 3.5% for low dose) [46]. The CONQUER trial evaluated 2.487 patients with BMI between 27–45 kg/m^2^ with two or more comorbidities. By ITT analysis, the results were similar, with loss of 10.4%, 8.4% and 1.8% for Qnexa^®^ high dose, medium dose and placebo, respectively. (PSWL of 8.6% for high dose and 6.6% for medium dose) [46]. There was significant improvement of a number of cardiovascular risk markers in patients using the medication, including: Waist circumference, systolic and diastolic BP and lipids in general. Significant improvements were reported in fasting blood glucose, HbA1c and HOMA index in diabetic patients for both doses.

The most common side effects, in order of frequency, were: dry mouth, paresthesias, constipation, upper respiratory tract infection, taste alteration and insomnia. The dropout rate ranged from 31 to 43% for patients taking Qnexa^®^ and 47% for the patients on placebo. Eighteen per cent of patients taking Qnexa^®^ high dose discontinued the study because of side effects compared to 9% for the placebo group. Clinically significant changes in cognitive function, depression and anxiety, or effects on psychomotor skills were not reported in greater frequence by patients taking the medication. It is unclear if the lower incidence of adverse effects with Qnexa^®^ compared to earlier studies involving topiramate is related to the use of lower doses of this drug or because of its SR formulation.

### 4.6. Pramlintide/Metreleptin

The control of energy homeostasis and body weight involves complex interactions between the CNS and peripheral neurohormonal signals from the adipose tissue, pancreas and GI tract [36,37,47].

These interactions include the integration of long-term adiposity signals, like insulin and leptin, and short-term satiety signals, such as amylin, peptide YY, incretins and cholecystokinin. Insulin and leptin show tonic secretion by the pancreas and adipose tissue, respectively, while the others are episodically secreted in response to the presence of food in the GI tract [48,49].

Amylin is a 37-amino acid peptide hormone co-secreted with insulin by pancreatic β-cells, which binds to receptors in the hindbrain, leading to increased satiety and a reduction in food intake [48]. In previous studies with obese patients on monotherapy, this hormone showed maintained and prolonged weight loss [49]. Pramlintide, an amylin analog, has shown to be effective in promoting weight loss in obese patients when used in monotherapy [50].

Leptin is a neurohormone secreted predominantly by adipocytes which bind to receptors in the hypothalamus, presenting a key role in the regulation of long-term energy homeostasis [34,47,48]. It is known that humans leptin-deficient present severe hyperphagia and morbid obesity and that the replacement of methyl-recombinant human leptin (metreleptin) in these patients leads to an almost complete reversal of this phenotype. However, in obese patients without leptin deficiency, the administration of this substance alone in monotherapy, even in high pharmacological doses, failed to produce substantial weight loss [51]. It is important to understand how these substances interact with one another, both in the dynamic weight loss phase and in the weight-reduced phase. The circulating levels of leptin drop rapidly after diet-induced weight loss, raising a series of counterregulatory hormonal, metabolic and autonomic responses, responsible for maintaining the initial weight [52]. This is one of the compensatory mechanisms which hinders the progression or even the maintenance of a determined reduction of body weight. Interestingly, it was demonstrated previously that restoration of basal leptin levels, through the replacement of metreleptin in humans undergoing diet-induced weight loss, helps to alleviate these counterregulatory responses of weight loss [53]. In this study, Rosenbaum *et al.* elegantly demonstrated that, after metreleptin administration, energy expenditure, skeletal muscle work efficiency, sympathetic nervous system tone, and circulating concentrations of thyroxine and triiodothyronine returned to pre–weight-loss levels [52]. 

Diet-induced leptin fall also contributed to reduce the anorexigenic effect of the satiety signals in the hindbrain. On the other hand, there is growing recognition that hindbrain signals may exert important effects on leptin signaling in the hypothalamus [36,47,48].

Preclinical studies in rats with diet-induced obesity showed that combined treatment with amylin and leptin led to a pronounced response and synergy in the reduction of food intake (up to 45%) and of body weight (up to 15%) [53].

It was proven that weigh loss was fat-specific and not accompanied by reductions of energy expenditure and fat oxidation normally seen with caloric restriction. These figures corroborated, therefore, the hypothesis that the combination of pramlintide with metreleptin would have a potential role in the treatment of obese humans.

The first clinical trial with this combination involved 177 obese patients with BMI between 30–35 kg/m^2^ (81%), or overweight patients with BMI between 27–30 kg/m^2^, with increased waist circumference (men > 102 cm, women > 88 cm) [48]. The study had a total duration of 24 weeks, with the first four weeks of titration of pramlintide (180 mcg bid in the first two weeks and 360 mcg bid in the following two, subcutaneously), in combination with a 40% calorie deficit diet. After this period, the patients who had lost from 2–8% of initial weight (n = 139) were randomly divided in a proportion of 1:2:2 to receive metreleptin, 5 mg bid, pramlintide, 360 mcg bid, or the combination. According to the authors, at the time of the study there were not sufficient placebo vials which explained the non-inclusion of this group. After 24 weeks, weight loss was significantly higher in the group receiving the combined treatment of pramlintide/metreleptin (−12.7%) than pramlintide (−8.4%) or metreleptin (−8.2%) alone. These findings were published in relation to the population which completed the study, and the authors did not disclose numerical data regarding the ITT analysis, albeit reported this difference to be significant. Although within normal range during screening, there was a trend towards improved levels of triglycerides, total cholesterol, LDL cholesterol, glycemia, insulinemia and insulin resistance measured by HOMA.

The commonest side effects reported were adverse reactions at the site of injection (pain, erythema, 58.9%) and nausea (12.5%). Other side effects were much less frequent, occurring in up to 5% of the cases, and included nasopharyngitis, headache, constipation, and diarrhea. Dropout rates were indifferent between the groups, ranging from 30–34%. One patient in each group left due to adverse reactions at the site of injection and one patient in the combined treatment group abandoned due to nausea. Four patients had already left the study due to nausea in the initial weeks of pramlintide titration. 

### 4.7. Pramlintide/Sibutramine or Phentermine

A randomized controlled trial compared the safety and efficacy of pramlintide alone or in combination with either oral sibutramine or phentermine [54]. Following a 1-week placebo lead-in, 244 obese or overweight, nondiabetic received placebo subcutaneously (sc) tid, or pramlintide sc, 120 mcg tid, either isolatedly or in association with sibutramine, 10 mg/d, or phentermine, 37.5 mg/d, for 24 weeks.

At the end of the study, weight loss was similar for the combinations of pramlintide/sibutramine (11.3 ± 1.2 kg) and pramlintide/phentermine (11.3 ± 0.9 kg) and significantly greater than that observed for pramlintide alone (3.6 ± 0.7 kg) and placebo (2.1 ± 0.9 kg). PSWL was 1.5% for pramlintide alone, 8.9% for pramlintide/sibutramine and 9.1% for pramlintide/phentermine [54]. Treatment was single-blind for subjects receiving sc medication only and open-label for subjects in the combination arms. Weight loss with both combinations seemed to be greater than that observed previously for the oral drugs in monotherapy, although the absence of such groups in this trial does not allow a direct comparison. Therefore, although this combination might become a novel promising tool of obesity pharmacotherapy, randomized controlled trials assessing this issue should be developed aiming a definitive answer.

### 4.8. Glucagon-Like Peptide 1/PYY 3-36

Glucagon-like peptide 1 (GLP-1) is a gut hormone rapidly secreted in response to food intake [55]. GLP-1 inhibits gastric emptying and the secretion of glucagon and stimulates glucose-dependent insulin secretion, lowering glycemia [37,55]. Peripheral administration of GLP-1 in obese diabetic subjects demonstrated a reduction in food ingestion [56]. A GLP-1 agonist, exenatide (Byetta^®^), when administered sc bid, leads to moderate and discreet weight loss in patients with T2DM [57]. Moreover, liraglutide, a GLP-1 homologue, when administered sc once a day, promoted weight loss and reduced obesity-related risk factors and prediabetes in obese subjects without type 2 diabetes [58]. Peptide YY (PYY 3-36) is gut hormone which is also secreted after a meal [37,59]. Intravenous infusion of PYY 3-36 has shown to produce a 30% reduction of food ingestion [60]. Some studies have also shown that combining oral administration of GLP-1, 2 mg, and PYY 3-36, 1 mg, may lead to a reduction of food intake [61].

## 5. Off-Label Combinations Used in Clinical Practice without Studies

### 5.1. Phentermine/5-HTP + Carbidopa

5-Hydroxytryptophan (5-HTP) is the precursor of serotonin [62] and is known, in high doses (900 mg/d), to induce weight loss, although with a high rate of side effects (mainly nausea, due to conversion to serotonin). Carbidopa inhibits the peripheral conversion of 5-HTP to serotonin, attenuating the side effects and allowing 5-HTP to reach the CNS, where it will be converted to serotonin [62]. There are no trials studying the combination of phentermine with 5-HTP/Carbidopa, but 20% of obesity specialists in the USA reported to use this combination in clinical practice [11], more than the use of sibutramine or orlistat. After this publication, one retrospective review of 22 patients sent by one of the members of the American Society of Bariatric Physicians showed that after six months of treatment with phentermine (with a weight loss of 16%), the addition of 5-HTP-Carbidopa resulted in an extra weight loss of 1% in the next six months, a reasonable result considering the expressive weight loss saw in the first six months [11]. Another clinical series involving 100 subjects showed 5% weight loss after four weeks using this combination and 18% after six months in patients who had not discontinued [63]. Randomized controlled studies are likely to be done in the following years, based on these data and the large number of prescriptions. 

### 5.2. Orlistat/Noradrenergic Drugs

The combination of orlistat with noradrenergic drugs is frequently used in clinical practice, albeit the safety and efficacy of this combination has not been assessed in randomized controlled studies. Considering they act on different pathways [4,5,6,13,14] and that the pharmodynamic characteristics of these medications do not imply a greater risk of side effects with their combination, the use of orlistat in doses of 240 to 360 mg/d before meals and catecholaminergic drugs in standard dosages, may result in an additive effect on weight loss, validating this combination for the treatment of obesity. Despite studies with catecholaminergic drugs have mainly been done for short periods of less than 24 weeks, we have recently shown that long-term use of diethylpropion can be safe and effective in a well-selected population.

### 5.3. Orlistat/Topiramate

Topiramate use as monotherapy for obesity treatment is not recommended as its usual side effects greatly reduce the tolerability and safety of the medication. However, for specific groups of patients, such as those who present associated conditions which indicate the need for treatment with topiramate (e.g., migraine, epilepsy or anxiety disorders), the combination of orlistat for an additive effect of weight loss seems reasonable, since they have distinct mechanisms of action and apparently there is no interaction between these two medications.

### 5.4. Orlistat/Antidepressants

Anxiety disorders, depression and eating disorders are considered risk factors for obesity. The treatment of these illnesses with antidepressants many times results in weight variation, depending on the drug class that is used [15,34,64,65]. For instance, tricyclic antidepressants and paroxetine may result in pronounced weight gain, while other selective serotonin reuptake inhibitors (e.g., fluoxetine and sertraline) and serotonin and noradrenaline reuptake inhibitors (e.g., duloxetine and venlafaxine) have shown to be neutral or even promote modest weight loss [15,65]. The use of these drugs can contraindicate the use of noradrenergic agents or sibutramine for the treatment of obesity, due to potentially hazardous interactions between the drugs (e.g., serotoninergic syndrome) as well as possible worsening of the underlying psychiatric disease. However, the addition of orlistat is not contraindicated and is a valid option for treating obesity in these conditions.

### 5.5. Sibutramine/Topiramate

Sibutramine and topiramate act on the CNS, through different mechanisms of action. Both led to sustained weight loss in studies carried out with the drugs in monotherapy [4,44]. Therefore, combination of both medications for the treatment of obesity seems rational. This has been performed in clinical practice, but randomized controlled studies have not been carried out that could confirm the safety and efficacy of this combination. It should be used with caution, individualized and should be tried exclusively in those patients experiencing failure of other existing therapies. We also routinely consider this combination in associated conditions which topiramate has proven to be effective, such as in patients suffering from migraine and BED [44,45]. It should be overemphasized that this combination should be used with caution in epileptic patients, since that seizures have been reported with sibutramine [66]. 

## 6. Conclusions

Obesity is a chronic, multifactorial disease, still very challenging for the clinician to treat. Lifestyle modifications, such as diet and physical activity, are very often difficult to achieve and maintain in the medium to long term. Medical therapy appear as an important treatment modality, but drugs most studied to date show modest results for sustained weight loss when compared with bariatric surgery, which is, however, still reserved for a selected group of patients. As the obesity epidemic progresses worldwide in an alarming scale, development of new therapeutic strategies, in which combination of drugs are included, is of crucial importance. 

Although combining drugs may increase the risk of side effects, the use of medications which act in different pathways, or which have synergistic actions, can help overcome regulatory mechanisms which prevent continued weight loss.

Most of the combinations used in clinical practice do not have, or have few studies which support them, but may be carefully used in clinical practice, customizing their use according to patients’ characteristics, while new studies or new drugs are not available.

It is encouraging that a number of drugs which individually lead to modest weight loss, have positive results when used in combination, even though currently released studies are in initial stages and/or still linked to pharmaceutical industries.

## References

[1] (2010). Global Strategy on Diet, Physical Activity and Health.

[2] Flegal K.M., Graubard B.I., Williamson D.F., Gail M.H. (2007). Cause specific excess deaths associated with underweight, overweight and obesity. J. Am. Med. Assoc..

[3] Douketis J.D., Macie C., Thabane L., Williamson D.F. (2005). Systematic review of long-term weight loss studies in obese adults: Clinical significance and applicability to clinical practice. Int. J. Obes. (Lond.).

[4] Rucker D., Padwal R., Li S.K., Curioni C., Lau D.C.W. (2007). Long term pharmacotherapy for obesity and overweight: Updated meta-analysis. Br. Med. J..

[5] Bray G.A. (2008). Lifestyle and pharmacological approaches to weight loss: Efficacy and safety. J. Clin. Endocrinol. Metab..

[6] Cercato C., Roizenblatt V.A., Leança C.C., Segal A., Lopes Filho A.P., Mancini M.C., Halpern A. (2009). A randomized double-blind placebo-controlled study of the long-term efficacy and safety of diethylpropion in the treatment of obese subjects. Int. J. Obes..

[7] Padwal R., Kezouh A., Levine M., Etminan M. (2007). Long-term persistence with orlistat and sibutramine in a population based cohort. Int. J. Obes..

[8] Sjöström L., Narbro K., Sjöström C.D., Karason K., Larsson B., Wedel H., Lystig T., Sullivan M., Bouchard C., Carlsson B., Bengtsson C., Dahlgren S., Gummesson A., Jacobson P., Karlsson J., Lindroos A.K., Lönroth H., Näslund I., Olbers T., Stenlöf K., Torgerson J., Agren G., Carlsson L.M., Swedish Obese Subjects Study (2007). Effects of bariatric surgery on mortality in Swedish obese subjects. N. Engl. J. Med..

[9] U.S. Food and Drug Administration (FDA) (2009). Follow-Up to the November 2009 Early Communication about an Ongoing Safety Review of Sibutramine, Marketed as Meridia.

[10] Halpern A., Pepe R.B., Monegaglia A.P., Beyruti M., de Melo M.E., Mancini M.C. (2010). Efficacy and tolerability of the association of sibutramine and orlistat for 6 months in overweight and obese patients. J. Obes..

[11] Hendricks E.J., Rothman R.B., Greenway F.L. (2009). How physician obesity specialists use drugs to treat obesity. Obesity (Silver Springs).

[12] Gadde K.M., Allison D.B. (2009). Combination pharmaceutical therapy for obesity. Expert Opin. Pharmacother..

[13] Munro J.F., MacCuish A.C., Wilson E.M., Duncan L.J.P. (1968). Comparison of continuous and intermittent anorectic therapy in obesity. Br. Med. J..

[14] Truant A.P., Olon L.P., Cobb S. (1972). Phentermine resin as an adjunct in medical weight reduction: A controlled randomized, double-blind prospective study. Curr. Ther. Res. Clin. Exp..

[15] Halford J.C.G., Harrold J.A., Boyland E.J., Lawton C.L., Blundell J.E. (2007). Serotonergic drugs: Effects on appetite expression and use for the treatment of obesity. Drugs.

[16] Guy-Grand B., Apfelbaum M., Crepaldi G., Gries A., Lefebvre P., Turner P. (1989). International trial of long-term dexfenfluramine in obesity. Lancet.

[17] Weintraub M., Hasday J.D., Mushlin A.I., Lockwood D.H. (1984). A double-blind clinical trial in weight control: Use of fenfluramine and phentermine alone or in combination. Arch. Intern. Med..

[18] Connolly H.M., Crary J.L., McGoon M.D. (1997). Valvular heart disease associated with fenfluramine-phentermine. N. Engl. J. Med..

[19] Astrup A., Toubro S., Christensen N., Quaade F. (1992). Pharmacology of thermogenic drugs. Am. J. Clin. Nutr..

[20] Ioannides-Demos L.L., Proietto J., McNeil J.J. (2005). Pharmacotherapy for obesity—review article. Drugs.

[21] Astrup A., Breum L., Toubro S., Hein P., Quaade F. (1992). The effect and safety of an ephedrine/caffeine compound compared to ephedrine, caffeine an placebo in obese subjects on an energy restricted diet. A double-blind trial. Int. J. Obes. Relat. Metab. Disord..

[22] Shekelle P.G., Hardy M.L., Morton S.C., Maglione M., Mojica M.A., Suttorp M.J., Rhodes S.L., Jungvig L., Gagné J. (2003). Efficacy and safety of ephedra and ephedrine for weight loss and athletic performance: A meta-analysis. J. Am. Med. Assoc..

[23] McBride B.F., Karapanos A.K., Krudysz A., Kluger J., Coleman C.I., White C.M. (2004). Electrocardiographic and hemodynamic effects of a multicomponent dietary supplement containing ephedra and caffeine: A randomized controlled trial. J. Am. Med. Assoc..

[24] Connoley I.P., Liu Y.L., Frost I., Reckless I.P., Heal D.J., Stock M.J. (1999). Thermogenic effects of sibutramine and its metabolites. Br. J. Pharmacol..

[25] Mancini M.C., Halpern A. (2008). Orlistat in the prevention of diabetes in the obese patient. Vasc. Health Risk Manag..

[26] Wadden T.A., Berkowits R.I., Wonble L.G. (2000). Effects of sibutramine plus orlistat in obese women following 1 year of treatment by sibutramine alone: A placebo controlled trial. Obes. Res..

[27] Kaya A., Aydin N., Topsever P., Filiz M., Öztürk A., Dağar A., Kılınç E., Ekmekcioglu C. (2004). Efficacy of sibutramine, orlistat and combination therapy on short-term weight management in obese patients. Biomed. Pharmacother..

[28] Sari R., Balci M.K., Cakir M., Altunbas H., Karayalcin U. (2004). Comparison of efficacy of sibutramine or orlistat *versus* their combination in obese women. Endocr. Res..

[29] Bray G.A. (2008). Is new hope on the horizon for obesity?. Lancet.

[30] Greenway F.L., Bray G.A. (2010). Combination drugs for treating obesity. Curr. Diab. Rep..

[31] Sari R., Balci M.K., Coban E., Yazicioglu G. (2004). Comparison of the effect of orlistat *vs* orlistat plus metformin on weight loss and insulin resistance in obese women. Int. J. Obes. Relat. Metab. Disord..

[32] Knowler W.C., Barrett-Connor E., Fowler S.E. (2002). Reduction in the incidence of type 2 diabetes with lifestyle intervention or metformin. N. Engl. J. Med..

[33] Contrave and Empatic. Orexigen Therapeutics, Inc.. http://www.orexigen.com/.

[34] Anderson J.W., Greenway F.L., Fujioka K. (2002). Bupropion SR enhances weight loss: A 48-week double-blind, placebo- controlled trial. Obes. Res..

[35] Lee M.W., Fujioka K. (2009). Naltrexone for the treatment of obesity: Review and update. Expert Opin. Pharmacother..

[36] Grill H.J. (2010). Leptin and the systems neuroscience of meal size control. Front Neuroendocrinol..

[37] Cummings D.E., Overduin J. (2007). Gastrointestinal regulation of food intake. J. Clin. Invest..

[38] Greenway F.L., Whitehouse M.J., Guttadauria M., Anderson J.W., Atkinson R.L., Fujioka K., Gadde K.M., Gupta A.K., O’Neil P., Schumacher D., Smith D., Dunayevich E., Tollefson G.D., Weber E., Cowley M.A. (2009). Rational design of a combination medication for the treatment of obesity. Obesity (Silver Springs).

[39] Padwal R. (2009). Contrave, a bupropion and naltrexone combination therapy for the potential treatment of obesity. Curr. Opin. Investig. Drugs.

[40] Greenway F.L., Dunayevich E., Tollefson G., Erickson J., Guttadauria M., Fujioka K., Cowley M.A., NB-201 Study Group (2009). Comparison of combined bupropion and naltrexone therapy for obesity with monotherapy and placebo. J. Clin. Endocrinol. Metab..

[41] Oommen K.J., Mathews S. (1999). Zonisamide: A new antiepileptic drug. Clin. Neuropharmacol..

[42] Gadde K.M., Franciscy D.M., Wagner H.R., Krishnan K.R. (2003). Zonisamide for weight loss in obese adults: A randomized controlled trial. J. Am. Med. Assoc..

[43] McElroy S.L., Kotwal R., Guerdjikova A.I., Welge J.A., Nelson E.B., Lake K.A., D’alessio D.A., Keck P.E., Hudson J.I. (2006). Zonisamide in the treatment of binge eating disorder with obesity: A randomized controlled trial. J. Clin. Psychiatry..

[44] Astrup A., Toubro S. (2004). Topiramate: A new potential pharmacological treatment for obesity. Obes. Res..

[45] Leombruni P., Lavagnino L., Fassino S. (2009). Treatment of obese patients with binge eating disorder using topiramate: A review. Neuropsychiatr. Dis. Treat..

[46] Vivus, Inc Qnexa Obesity.

[47] Woods S.C., D´Alessio D.A. (2008). Central Control of Body Weight and Appetite. J. Clin. Endocrinol. Metab..

[48] Ravussin E., Smith S.R., Mitchell J.A., Shringarpure R., Shan K., Maier H., Koda J.E., Weyer C. (2009). Enhanced weight loss with pramlintide/metreleptin: An integrated neurohormonal approach to obesity pharmacotherapy. Obesity (Silver Spring).

[49] Smith S.R., Aronne L.J., Burns C.M., Kesty N.C., Halseth A.E., Weyer C. (2008). Sustained weight loss following 12-month pramlintide treatment as an adjunct to lifestyle intervention in obesity. Diabetes Care.

[50] Heymsfield S.B., Greenberg A.S., Fujioka K., Dixon R.M., Kushner R., Hunt T., Lubina J.A., Patane J., Self B., Hunt P., McCamish M. (1999). Recombinant leptin for weight loss in obese and lean adults: A randomized, controlled, dose-escalation trial. J. Am. Med. Assoc..

[51] Rosenbaum M., Goldsmith R., Bloomfield D., Magnano A., Weimer L., Heymsfield S., Gallagher D., Mayer L., Ellen M., Leibel R.L. (2005). Low-dose leptin reverses skeletal muscle, autonomic, and neuroendocrine adaptations to maintenance of reduced weight. J. Clin. Invest..

[52] Trevaskis J.L., Coffey T., Cole R., Lei C., Wittmer C., Walsh B., Weyer C., Koda J., Baron A.D., Parkes D.G., Roth J.D. (2008). Amylin-mediated restoration of leptin responsiveness in diet-induced obesity: Magnitude and mechanisms. Endocrinology.

[53] Aronne L.J., Halseth A., Burns C., Miller S., Shen L.Z. (2010). Enhanced weight loss following co-administration of pramlintide with sibutramine or phentermine in obese subjects. Obesity (Silver Spring).

[54] Turton M.D., O’Shea D., Gunn I., Beak S.A., Edwards C.M.B., Meeran K., Choi S.J., Taylor G.M., Heath M.M., Lambert P.D., Wilding J.P.H., Smith D.M., Ghatei M.A., Herbert J., Bloom S.R. (1996). A role for glucagon-like peptide-1 in the central regulation of feeding. Nature.

[55] Zander M., Madsbad S., Madsen J.L., Holst J.J. (2002). Effect of 6-week course of glucagon-like peptide-1 on glycaemic control, insulin sensivity, and β-cell function in type 2 diabetes: A parallel group study. Lancet.

[56] DeFronzo R.A., Ratner R.E., Han J., Kim D.D., Fineman M.S., Baron A.D. (2005). Effects of exenatide (exendin-4) on glycemic control and weight over 30 weeks in metformin-treated patients with type 2 diabetes. Diabetes Care.

[57] Astrup A., Rössner S., van Gaal L., Rissanen A. (2009). NN8022-1807 Study Group. Effects of liraglutide in the treatment of obesity: A randomised, double-blind, placebo-controlled study. Lancet.

[58] Adran T.E., Ferri G.L., Bacarese-Hamilton A.J., Fuessl H.S., Polak J.M., Bloom S.R. (1985). Human distribution and release of a putative new gut hormone, peptide YY. Gastroenterology.

[59] Batterham R.L., Cohen M.A., Ellis S.M., Le Roux C.W., Withers D.J., Frost G.S., Ghatei M.A., Blook S.R. (2003). Inhibition of food intake in obese subjects by peptide YY 3-36. N. Engl. J. Med..

[60] Emisphere Technologies, Inc (2009). Emisphere Technologies Reports Encouraging Data from Independent Clinical Study Assessing the Effects of Oral GLP-1 e PYY3-36, Combined with Eligen.

[61] Turner E.H., Loftis J.M., Blackwell A.D. (2006). Serotonin a la carte: Supplementation with the serotonin precursor 5-hydroxytryptophan. Pharmacol. Ther..

[62] Rothman R.B. (2009). Treatment of obesity with “combination” pharmacotherapy. Am. J. Ther..

[63] Jackson C.W., Cates M., Lorenz R. (2010). Pharmacotherapy of eating disorders. Nutr. Clin. Pract..

[64] Berkowitz R.I., Fabricatore A.N. (2005). Obesity, psychiatric status, and psychiatric medications. Psychiatr. Clin. North Am..

[65] Huang B.C., Liou H.H. (2009). Sibutramine-induced recurrent seizures. Epilepsy Behav..

